# Application of RNA processing factors for predicting clinical outcomes in colon cancer

**DOI:** 10.3389/fgene.2022.979001

**Published:** 2022-09-23

**Authors:** Liujin Hou, Fan Huang, Guanghou Chen, Jian Qiu, Yuyao Liu, Hongchuan Zhao, Zhengguang Wang

**Affiliations:** Department of General Surgery, The First affiliated Hospital of Anhui Medical University, Hefei, Anhui, China

**Keywords:** colon cancer, prediction model, prognosis, RNA processing factors, GEO database, TCGA data base

## Abstract

**Background:** Colon cancer is the fifth most common cause of cancer-related death worldwide, and despite significant advances in related treatment, the prognosis of colon cancer patients remains poor.

**Objective:** This study performs systematic bioinformatics analysis of prognostic-associated RNA processing factor genes in colon cancer using the Cancer Related Genome Atlas database to explore their role in colon carcinogenesis and prognosis and excavate potential therapeutic targets.

**Methods:** Data sets of colon cancer patients were obtained from GEO and TCGA databases. Univariate cox analysis was performed on the GSE39582 training set to identify prognosis-associated RNA processing factor genes and constructed a muticox model. The predictive performance of the model was validated by Correlation curve analysis. Similar results were obtained for the test dataset. Functional analyses were performed to explore the underlying mechanisms of colon carcinogenesis and prognosis.

**Results:** A constructed muticox model consisting of βi and prognosis-related RNA processing factor gene expression levels (Expi) was established to evaluate the risk score of each patient. The subgroup with a higher risk score had lower overall survival (OS), higher risk factor, and mortality. We found that the risk score, age, gender, and TNM Stage were strongly associated with OS, and the 13-gene signature as an independent prognostic factor for colon cancer. The model has good accuracy in predicting patient survival and is superior to traditional pathological staging.

**Conclusion:** This study proposes 13 RNA processing factor genes as a prognostic factor for colon cancer patients, which can independently predict the clinical outcome by risk score. The gene expression profile in this model is closely related to the immune status and prognosis of colon cancer patients. The interaction of the 13 RNA processing factor genes with the immune system during colon carcinogenesis provides new ideas for the molecular mechanisms and targeted therapies for colon cancer.

## Key messages

1. 13 RNA processing factor genes as a prognostic factor for colon cancer patients.
2. The gene expression profile is closely related to the immune status and prognostic survival of patients.
3. Immune system are involved in the interaction of the 13 RNA processing factor genes.

## Introduction

Colon cancer is the fourth most frequently diagnosed cancer and the fifth leading cause of cancer-related death globally, accounting for almost 1.2 million new cases and 0.8 million deaths per year (Siegel et al., 2020; Sung et al., 2021). Owing to the absence of early symptoms, most patients diagnosed with colon cancer are at an advanced stage. Surgical resection is currently the primary therapy for colon cancer, which is prone to recurrence and metastasis after resection, dramatically decreasing the survival time of patients (Siegel et al., 2017; Han et al., 2019; Jung et al., 2021; Le et al., 2021). Despite advances in the diagnosis and treatment of colon cancer in recent years, the low survival time, high recurrence rate, and poor prognosis are still challenging (Yang et al., 2019; Cerrito and Grassilli, 2021; Hoehn et al., 2021). The clinicopathologic staging system remains the gold standard for prognosis prediction and therapeutic decision-making in colon cancer. However, people with the same stage and therapy might have quite varied outcomes because of the high heterogeneity. As a result, it is critical to explore the molecular mechanisms behind colon cancer’s occurrence and progression in detail to reveal novel prognostic biomarkers and therapeutic targets.

Studies have shown that prognosis-associated RNA processing factor genes of human play an essential role in tumorigenesis and progression (Obeng et al., 2019; Lou et al., 2021). Thus, there is of great importance to actively search for relevant RNA processing factor genes that predict the prognosis of colon cancer patients to improve the curative effect and prognosis of patients. For instance, it has been shown that the expression of ubiquitination-related RNA processing factor genes in cancer tissues is closely related to the prognosis of patients with colon cancer (Ishii et al., 2014; Sanchez-Jimenez et al., 2015; Hopkins et al., 2016; Wurth et al., 2016). The construction of a systematic model of prognosis-related RNA processing factor gene expression levels facilitates the identification of concrete mechanisms and therapeutic targets of colon cancer pathogenesis, which carry great significance for precise therapy and improvement of patient prognosis.

In recent years, tumor survival models based on the expression of prognosis-related RNA processing factor genes have been established to assess prognosis and screen therapeutic targets, but it has not been reported in colon cancer (Li et al., 2020a; Li et al., 2020b). In this study, RNA processing factor genes associated with colon cancer were obtained from the Cancer Genome Atlas (TCGA) database, and a muticox model consisting of prognosis-associated RNA processing factor gene expression levels and their corresponding coefficients was established. The 13 gene signature was found to be an independent prognostic factor for colon cancer after multifactorial analysis, and also had good accuracy for predicting patient survival, even better than conventional pathological staging. In addition, further investigation on the interaction between these genes and the immunity will contribute to the discovery of potential molecular mechanisms and therapeutic targets of colon cancer.

## Methods

### Data retrieval and preprocessing

First, we obtained mRNA expression information of colon cancer from GEO (Gene Expression Omnibus) database and TCGA database and excluded patients with incomplete survival data. RNA-seq data were merged and standardized with “limma” package and combat algorithm. RNA processing factor genes were obtained from the AmiGO database.

### Identification of prognosis-related RNA processing factor genes and assessment of their value

We used the “survival” package to perform univariate cox analysis on the GSE39582 training set to identify prognosis-related RNA processing factor genes. To clarify the value of prognosis-related RNA processing factors further, we performed unsupervised consensus clustering to identify new subtypes using the ConsensusClusterPlus R package and performed Kaplan-Meier survival analysis and log-rank tests on the subtypes using the “survival” and “survminer” packages. Stromal and immune cells in malignant tissues were estimated using the Estimate algorithm, and the stromal score, immune score, and ESTIMATE score were calculated for the different molecular subtypes (Yoshihara et al., 2013). Stromal and immune cells in malignant tissues were estimated using the Estimate algorithm, and the stromal score, immune score, and ESTIMATE score were calculated for the different molecular subgroups. The abundance of immune cells in different molecular subgroups was evaluated with MCP-Counter. Although the MCP-Counter score does not represent the actual proportion of each immune cell subpopulation in the tumor tissue, it has some numerical advantages in downstream statistical analysis. In the MCP-Counter method, the abundance of the 10 immune cells was expressed as the log2 geometric mean of the transcriptional markers of these immune cells, called the MCP-Counter score.

### Construction and evaluation of prognostic models

In the training dataset, “glmnet” and “survival” packages were used to further screen prognosis-associated RNA processing factor genes. Then a muticox model consisting of prognosis-associated RNA processing factor gene expression levels (Exp) and their corresponding coefficients (β) was constructed with Risk score = ∑(β_1_*Exp_1_+β_2_* Exp_2_ +β_3_* Exp_3_+⋯+β_n_* Exp_n_) to evaluate the risk score of each patient. 70% of the patients were randomized into the training group, and the remaining 30% were in the test group. We divided patients into high-risk and low-risk groups using the median risk score as the cut-off value. The overall survival (OS) between the high-risk and low-risk groups was compared by Kaplan—Meier survival analysis and log-rank test. The model’s predictive effect was evaluated using receiver operator characteristic (ROC) curve analysis. Survival risk curves and scatter plots were used to illustrate the risk scores and survival status of each sample. The samples from the TCGA database were set as a validation cohort also for the above analysis to validate the performance of the constructed prediction model in predicting survival. In addition, we assessed the prognostic significance of risk scores and clinical variables such as age, sex, and T, N, and M staging by univariate and multivariate Cox regression analyses on the training set, described the relative risk in terms of hazard ratio (HR) and 95% confidence interval (CI), and used decision curve analysis (DCA) to determine whether the model could benefit patients.

### Application model

To facilitate the better application of our model by clinicians, we constructed the nomogram jointly with age, sex, and T, N, and M staging and evaluated the predictive effect of the nomogram using C-index, ROC curve, and calibration curve analysis. Notably, since chemotherapy is a common treatment for colon cancer, we used the GDSC (Genomics of Drug Sensitivity in Cancer) database to calculate the difference in the IC50 (half maximal inhibitory concentration) of chemotherapy drugs between patients in the high- and low-risk groups, with a smaller IC50 implying greater sensitivity to the drug. In addition, we used the cellminer database to calculate the correlation between the genes in the model and the Z score value of chemotherapeutic drug sensitivity; a higher Z score value means greater sensitivity to the drug.

### Exploring potential mechanisms

ssGSEA (single sample GSEA) is an implementation method proposed mainly for the single sample that cannot do GSEA and is similar to GSEA in principle. We calculated the immune cell and function gene set scoring in each sample and performed survival analysis by the ssGSEA method, and the difference analysis was performed between high-risk and low-risk groups. We also performed the differential analysis of genes in the model to clarify their differential expression in high and low-risk groups. In addition, we evaluated the differences of risk scores in microsatellite instability groupings. A gene set enrichment study was performed between the high- and low-risk groups to elucidate the potential biological mechanisms and signaling pathways associated with the 13-gene signature.

### Immunohistochemistry staining

Paraffin-embedded cancer and adjacent normal tissues were cut into 5-μm thick tissue sections and slides prepared using standard techniques. The sections were deparaffinized and rehydrated with graded alcohol, and then antigens were retrieved by heating in citric acid (pH6.0) buffer in a microwave oven. 3% hydrogen peroxide and 3% BSA were used to quench endogenous peroxidase activity and reduce non-specific binding respectively. According to the instruction, anti-BICD1 (dilution 1:100, HPA041309, Sigma-Aldrich) were incubated on the sections overnight at 4°C. Then, the sections were incubated with a horseradish peroxidase conjugated secondary antibody, and staining was visualized using DAB color developing solutions. At last, the sections were counterstained with hematoxylin and mounted with mounting medium.

### Statistical analysis

All statistical analyses were performed by R version 4.0.4 (Institute of Statistics and Mathematics, Vienna, Austria, https://www.r-project.org), taking the mean of duplicate values, removing missing values, and RNA-seq data were combined and standardized by the R package “limma.” Correlations were determined using Spearman correlation analysis, clinical variables were compared using Wilcoxon test and *t*-test, survival status was assessed by Cox regression analysis, Knowledge management analysis was performed using the R package “survival” and “survminer,” OS was generated using the Kaplan-Meier method and assessed by log-rank test, and two-tailed *p* < 0.05 was considered statistically significant. The sensitivity and specificity of the model were assessed using ROC curves from the R package “survivalROC,” and the heat map was presented through the R package “heat map.” In addition, we validated the confidence of the model using the test dataset and the whole dataset, using hazard ratios (HRs) and 95% confidence intervals (CIs) to describe the relative risk.

## Results

### Data retrieval and pre-processing

First, we downloaded the GSE39582 dataset from the GEO database to obtain gene expression data and related survival information of 523 colon cancer patients. During further validation, HTSeq-FPKM expression information of colon cancer was obtained from the TCGA database, and 372 colon cancer patients with complete follow-up information were included in the training set after excluding patients with incomplete survival information. The RNA-seq data were merged and normalized using the “limma” package processing. 929 RNA-processing factor genes were obtained from the AmiGO database, and 806 previously reported human RNA-processing genes were included in both the GSE39582 database and TCGA dataset.

### RNA processing factor genes could classify colon cancer patients into two significantly distinguishable subtypes

**FIGURE 1:**
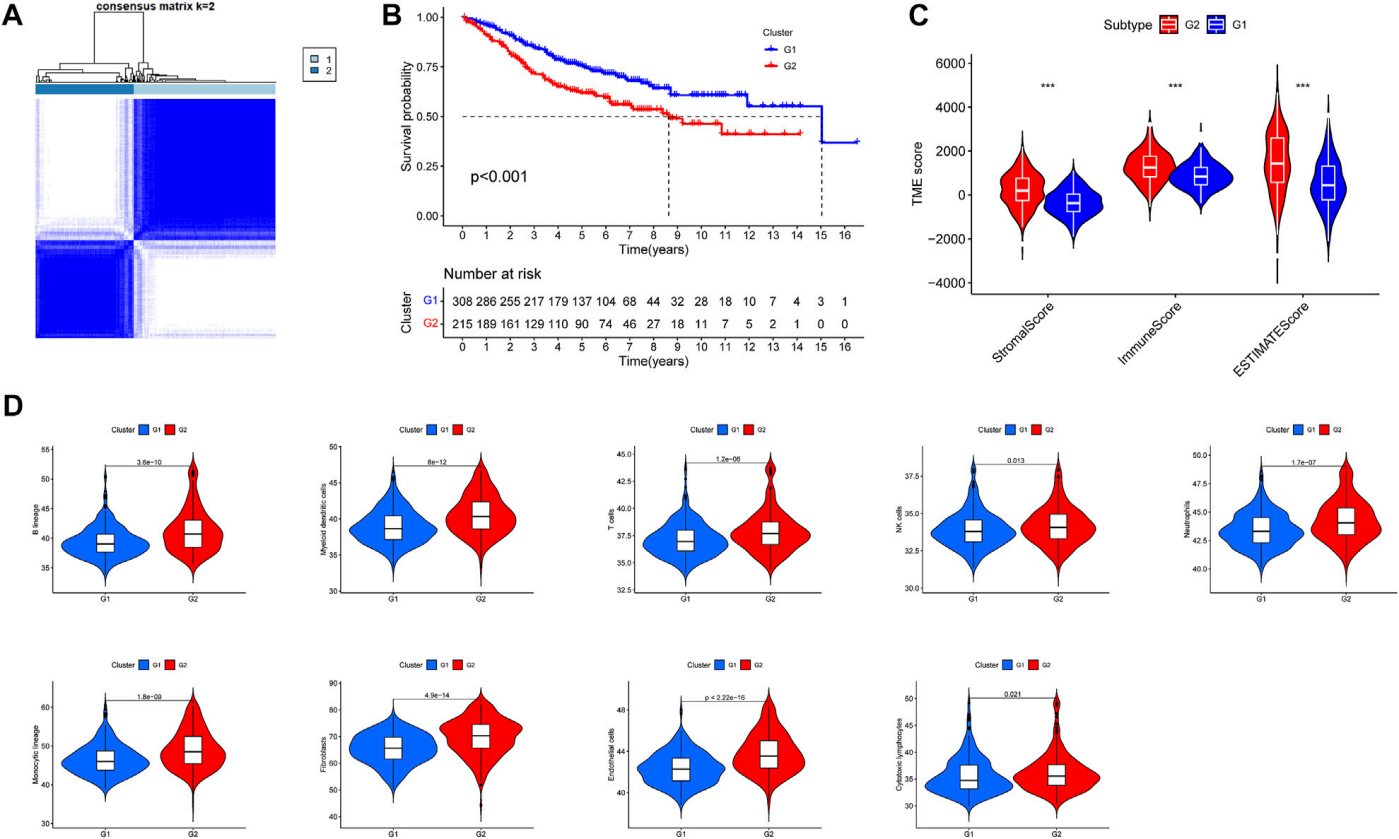
Subgroups of COAD (Colon Adenocarcinoma) defined by prognosis-associated RNA processing factor genes. **(A)** The TCGA cohort’s consensus score matrix for all samples when *K* = 2. When two samples had a higher consensus score in distinct interactions, they were more likely to be clustered together. **(B)** OS curves based on COAD patients from the TCGA cohort for the two RNA processing factor clusters. **(C)** Comparations between the two subgroups in terms of stromal score, immune score, and ESTIMATE score in tumor tissues. **(D)** Comparations between the two subgroups in abundance of immune filtrating cells in tumor tissues.

Univariate cox analysis based on the training group obtained 80 prognosis-related RNA processing factor genes (*p* < 0.05) and could classify colon cancer patients into two subgroups, G1 and G2 (Figure 1A). Survival analysis revealed that patients in the G1 group had a better prognosis (Figure 1B). Tumor microenvironment analysis suggested that the stromal, immune, and ESTIMATE scores were lower in the G1 group than in the G2 group (Figure 1C). Immune cell infiltration analysis showed a difference in a large number of immune cells between the G1 and G2 subgroups, and the level was lower in the G1 group (Figure 1D). This implies that there may be an immune barrier mediating immune escape in the G2 group, which we hypothesize is the reason for the poor prognosis in the G2 group. It also implies that the subtypes we identified are highly distinguishable and that prognosis-related RNA processing factor genes are of great value to explore.

### The 13-gene signature is an independent prognostic factor for colon cancer

**FIGURE 2:**
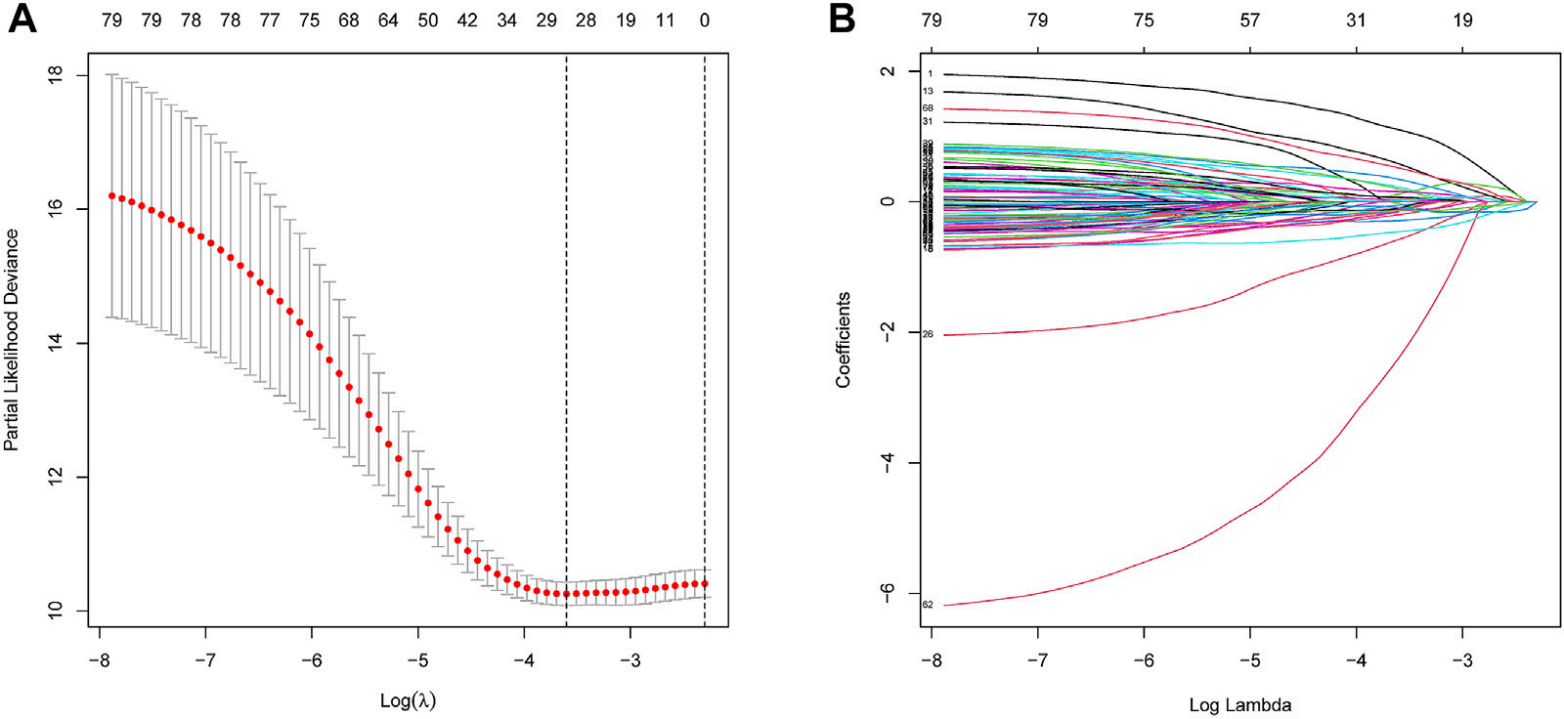
Construction of the RNA processing factors related risk signature model. **(A,B)** Partial likelihood deviance of variables revealed by the Lasso regression model. The red dots represented the partial likelihood of deviance values, the gray lines represented the standard error (SE), the two vertical dotted lines on the left and right represented optimal values by minimum criteria and 1-SE criteria, respectively.

**FIGURE 3:**
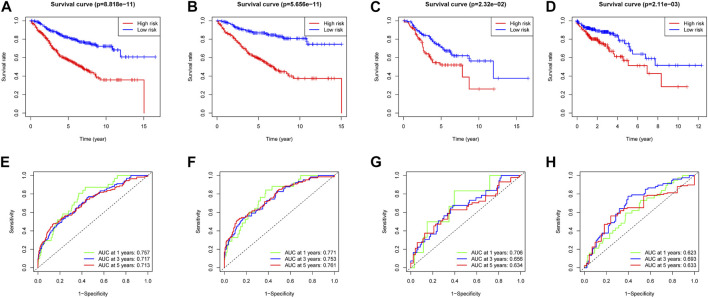
Survival analysis and ROC analysis of two risk groups of the 13-gene signature in training cohort **(A,E)**, testing cohort **(B,F)**, all patients **(C,G)**, and validation cohort **(D,H)**.

**FIGURE 4:**
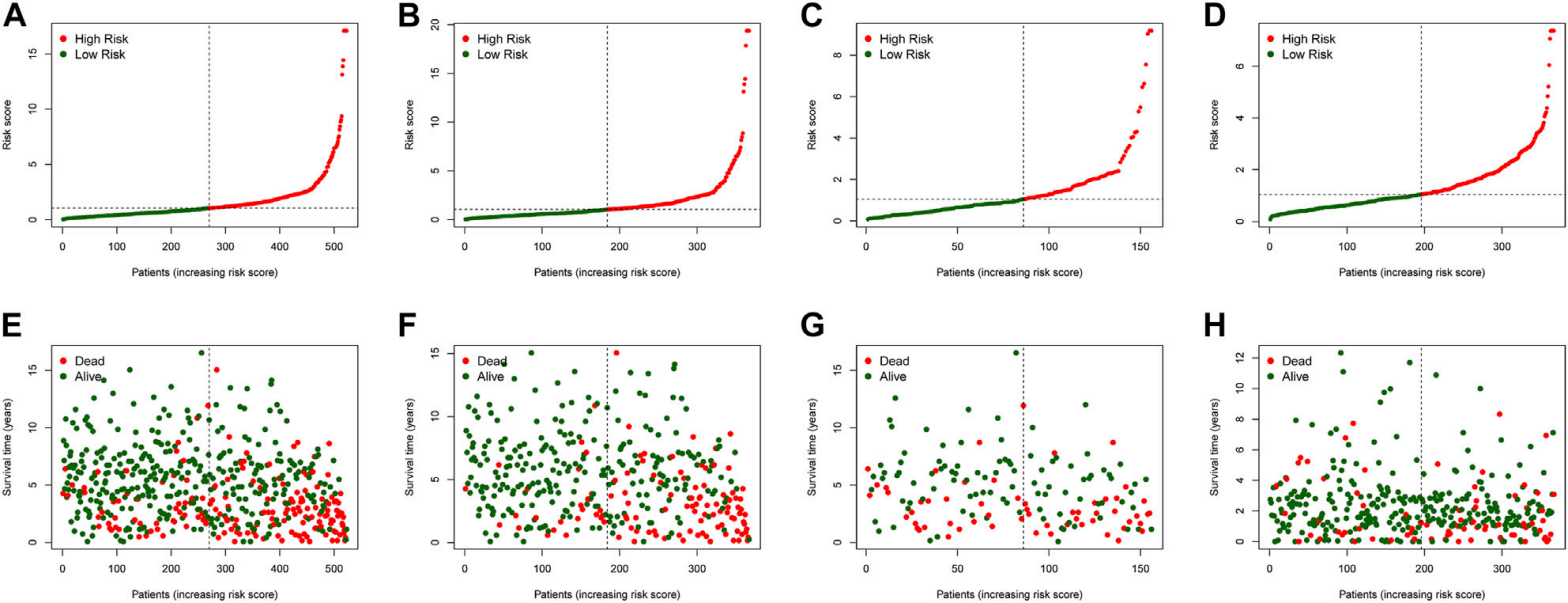
The risk score distribution and COAD cancer patients’survival status in the training cohort **(A,E)**, test cohort **(B,F)**, all patients **(C,G)**, and validation cohort **(D,H)** based on the risk score of the 4-gene signature.

**FIGURE 5:**
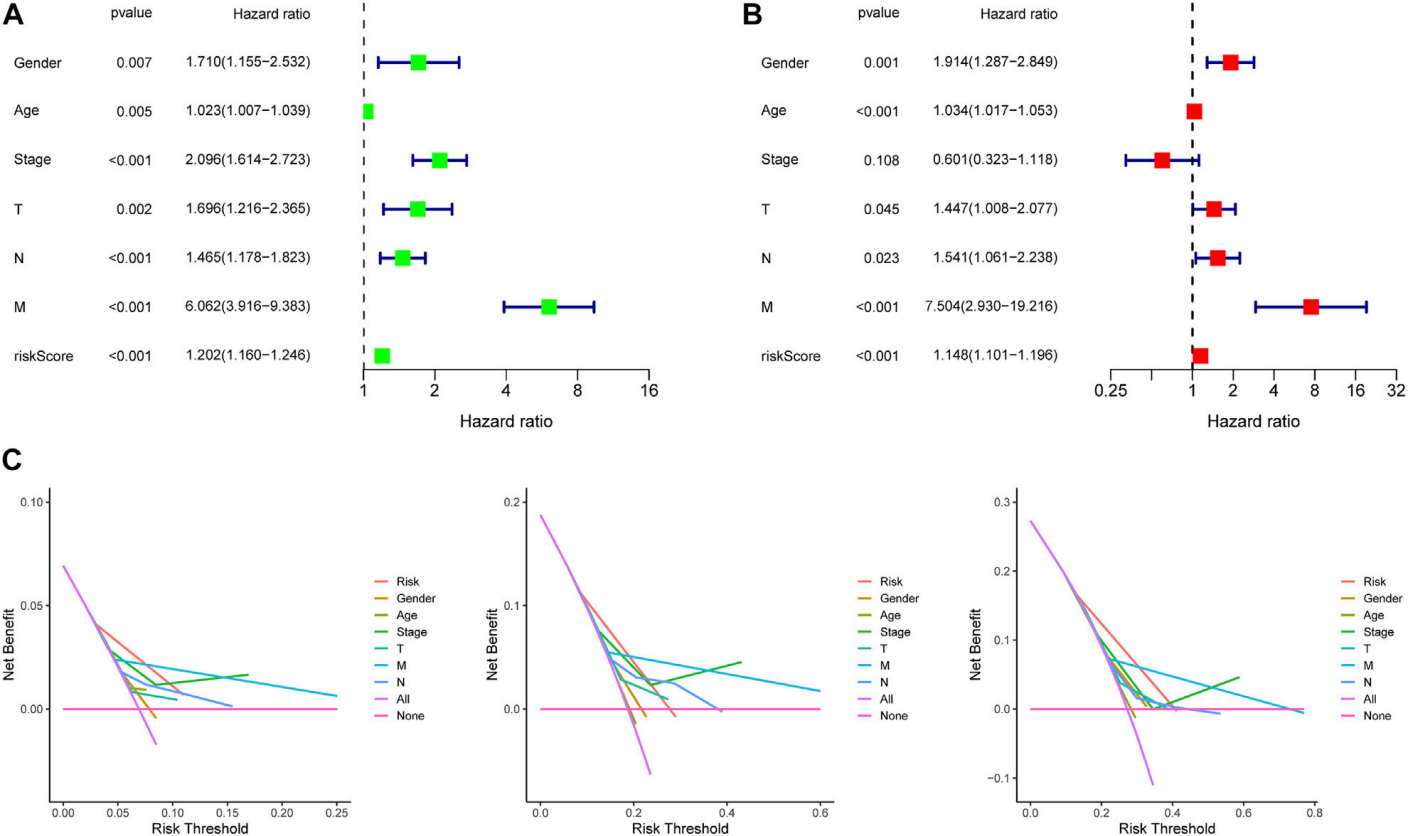
Independence detection and decision curve analysis of the constructed risk prediction model. **(A,B)** Independent prognostic-related factors screened out by COX regression analysis. **(C)**. The *y*-axis measures the net benefit. The red line represents the constructed risk prediction model. The purple line represents the assumption that all the patients were predicted using none indicators. The blue line represents the assumption that the patients were predicted using all indicators. The decision curve showed that using this constructed risk prediction model in the current study to predict COAD patients’ survival risk adds more benefit than the indicator-all-patients scheme or the indicator-none scheme.

In the training dataset, 13 prognosis-related RNA processing factor genes were further extracted by using the “glmnet” and “survivor” packages to filter and remove collinearity. Then, a muticox model consisting of prognosis-associated RNA processing factor gene expression levels and their corresponding coefficients was constructed to evaluate the risk score of each patient. The risk score= (CLK3*1.47577934073505) + (SNRPF*-0.498316806572549) + (CELF1*1.2745982697241) + (WDR43*-0.573978763865487) + (DEDD2*0.642794727626439) + (CMTR1*-1.24415055820031) + (SNRPA1*0.80184898855264) + (GTF2H5*0.699623576827454) + (FTSJ3*-0.576267186683901) + (ADAD1*-5.01566990560331) + (HNRNPUL1*-0.628475085028677) + (BICD1*1.07079849381416) + (THUMPD3*-0.551211262513779). We used the 13-gene signature risk score to quantify patients’ risk and used the median value as the cutoff (Figure 2). The results showed that the OS of the low-risk group was significantly better than that of the high-risk group, and the ROC curve analysis suggested an excellent sensitivity and specificity (Figure 3). Risk curves and scatter plots showed that patients in the high-risk group had higher hazard ratios and mortality rates than those in the low-risk group, and similar results were obtained in the test set (Figure 4). Univariate Cox regression showed that risk score and age, sex, and T, N, M stage were strongly associated with OS, and multivariate Cox analysis found that 13-gene signature was an independent prognostic factor for colon cancer (*p* < 0.001) (Figures 5A,B). DCA revealed that using our model to predict patients’ survival can make benefits (Figure 5C).

### The 13-gene signature has significant application value

**FIGURE 6:**
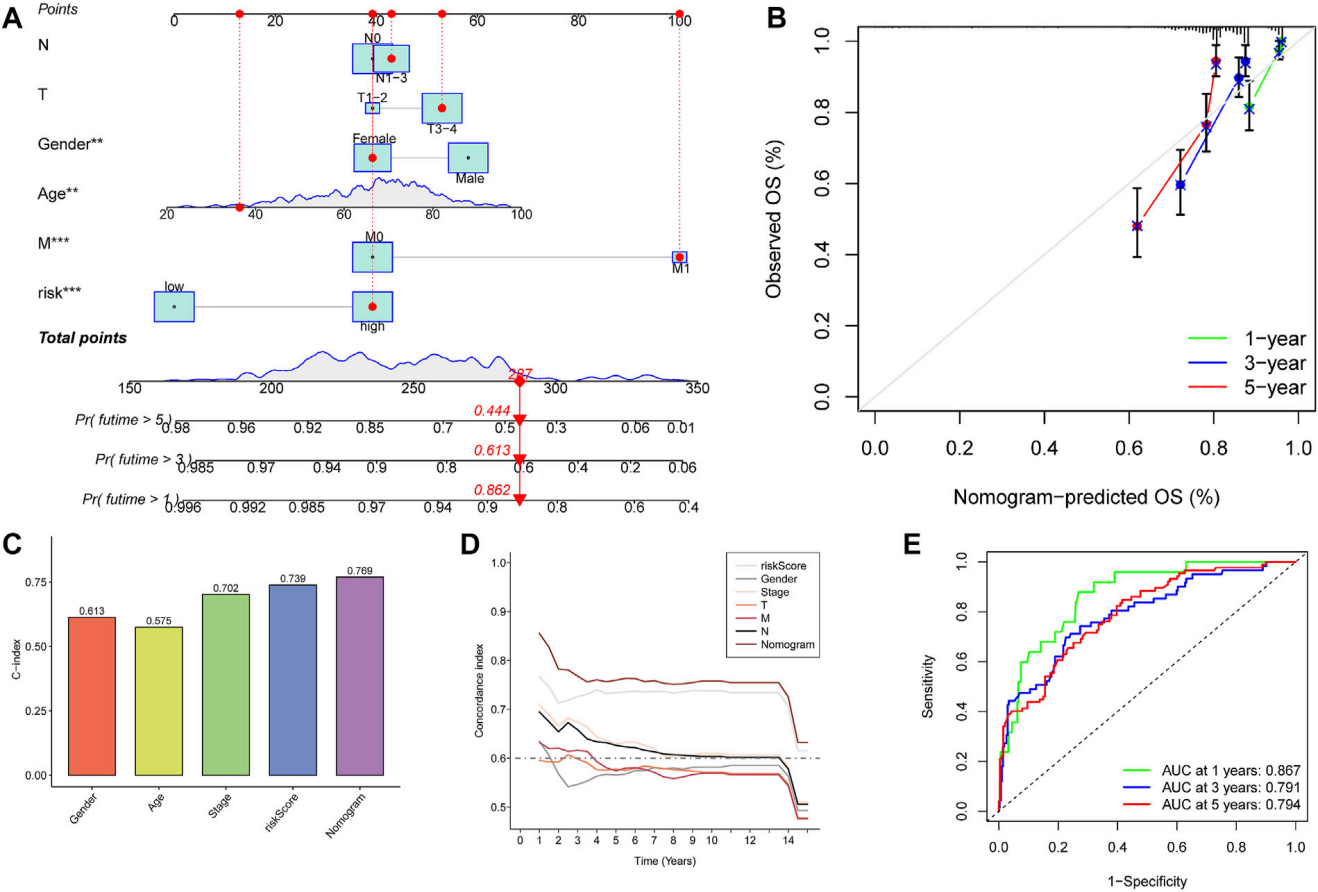
Construction and calibration of nomogram. **(A)** Nomogram integrating risk and clinical characteristics. **(B)** Calibration of the nomogram at 1-y, 3-y, and 5-y survival in the TCGA cohort. **(C,D)** C-index estimates the probability that the predicted results are consistent with the actual observed results. **(E)** Time-dependent ROC curve of the constructed nomogram model.

**FIGURE 7:**
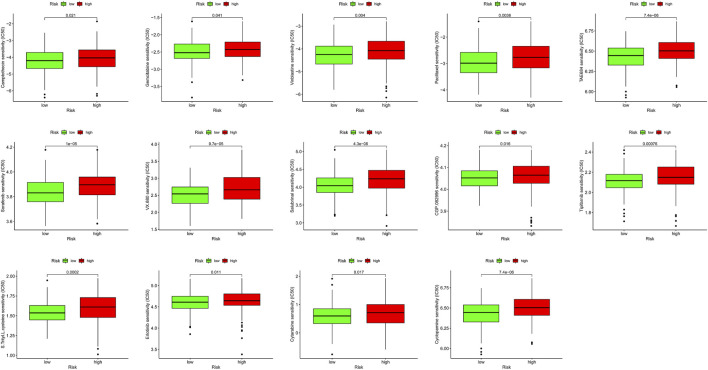
Immunotherapeutic and chemotherapeutic responses in high- and low-risk patients with COAD.

**FIGURE 8:**
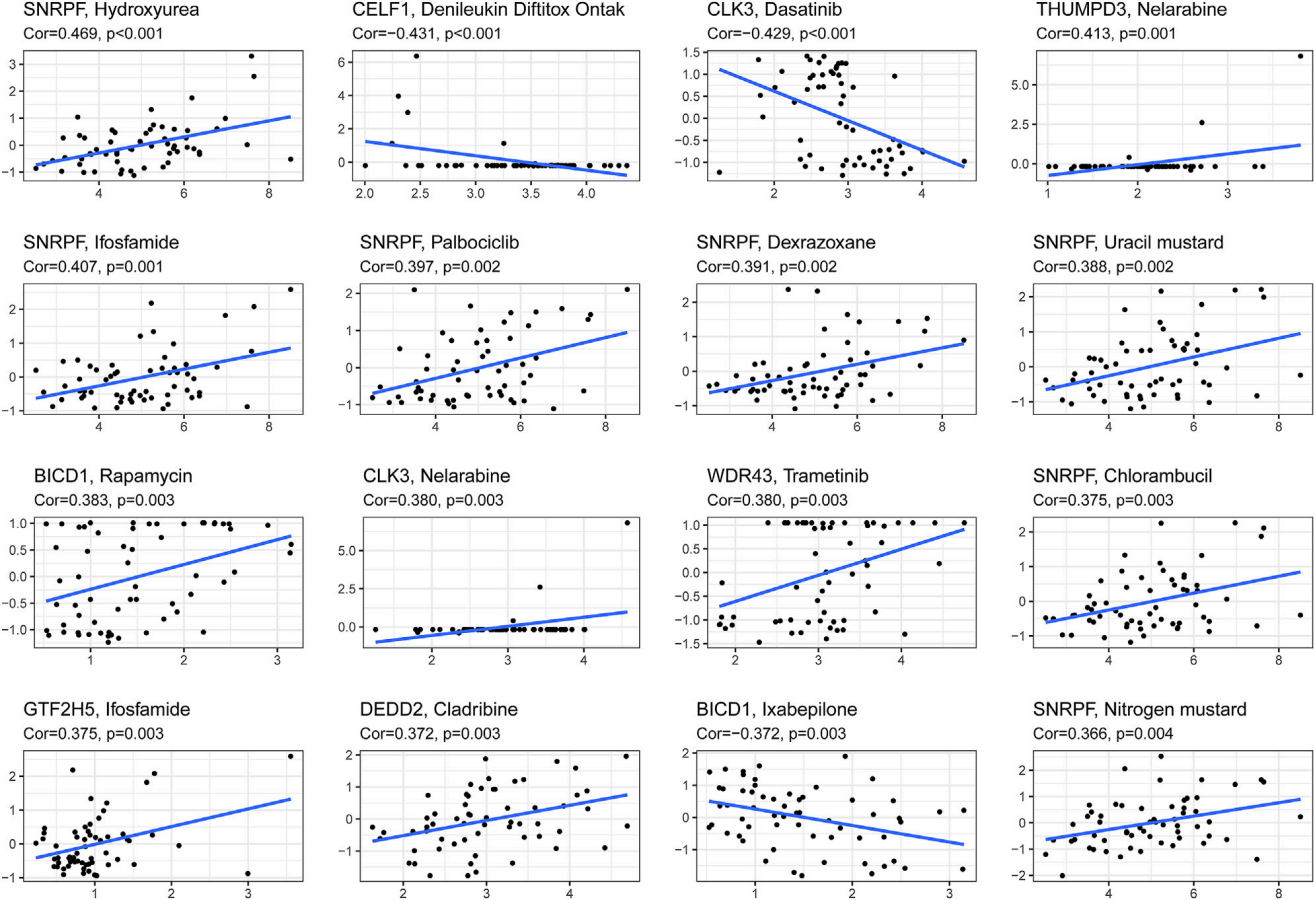
The correlation between genes in the signature and z-score values of chemotherapeutic drug sensitivity was calculated based on cellminer database.

We combined clinical traits to construct a line graph to determine the prognosis of patients more accurately, and the calibration curve and ROC suggest that our model has good calibration and predictive performance. It predicts patient survival better than conventional pathology and is a good complement to conventional pathology (Figure 6). In the drug sensitivity analysis, we found differences in the sensitivity of many chemotherapeutic drugs in high and low-risk groups (Figure 7). The genes in the model were moderately correlated with a large number of drugs (Figure 8). This implies that our signature can guide clinical drug use with significant application.

### Immune escape and mismatch repair are risk factors for poor prognosis in colon cancer patients

**FIGURE 9:**
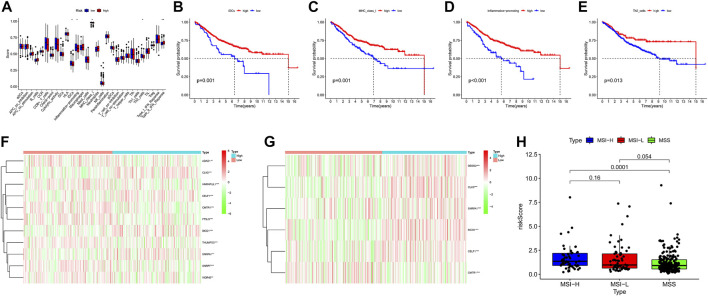
Degree of differentiation of the model. **(A)** Boxplots were used to display the expressions difference of Immune function score quantified by ssGSEA. ∗*p* < 0.05; ∗∗*p* < 0.01; ∗∗∗*p* < 0.001. **(B–E)** Survival analysis of two group divided by the median of Immune function score. **(F,G)**Heatmap display the expressions difference of gene mRNA in the 13 gene signature. **(H)** Rank-sum test was used to identify risk scores differences in microsatellite instability.

**FIGURE 10:**
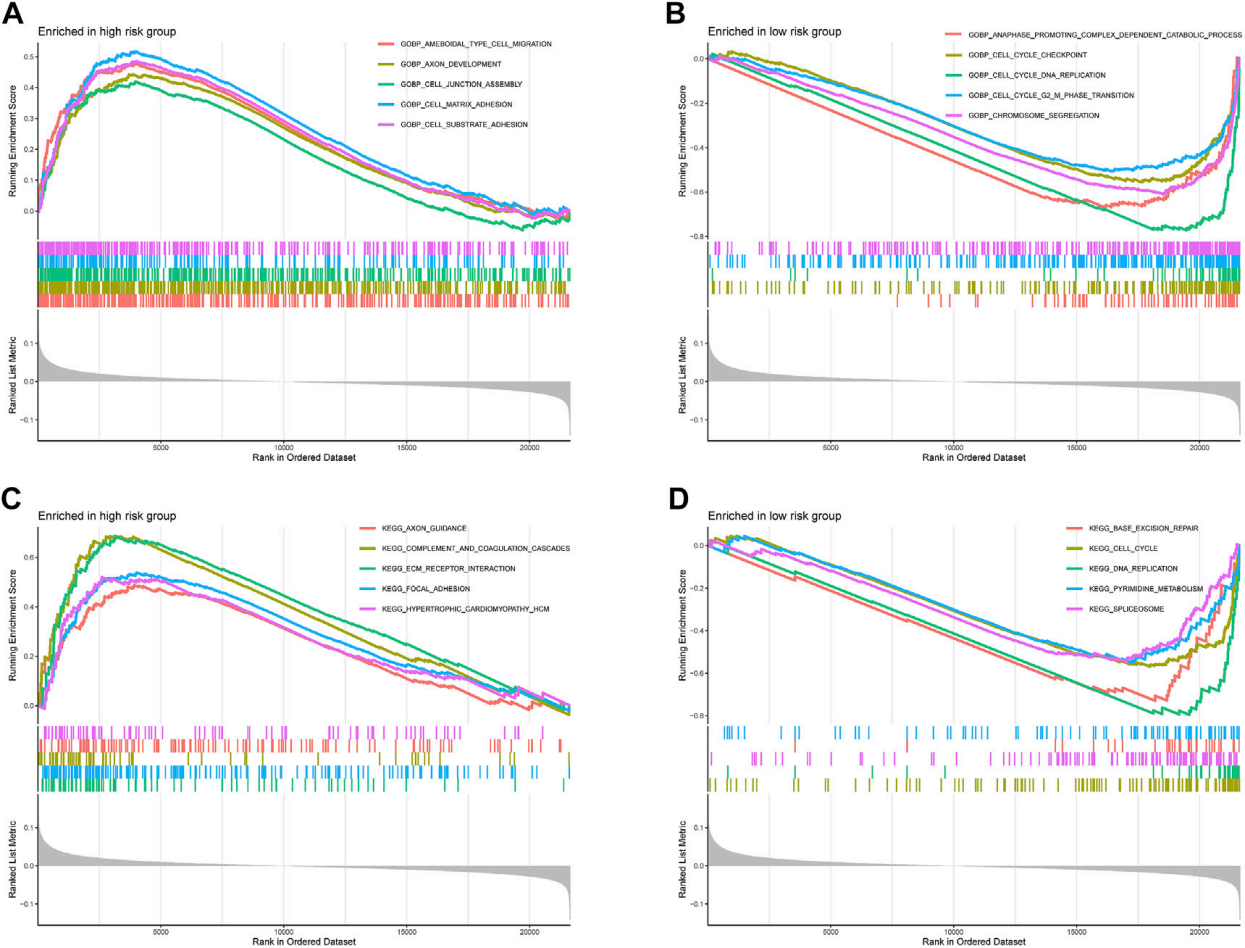
Functional enrichment analyses. **(A,B)** Gene Ontology and **(C,D)** Kyoto Encyclopedia of Genes and Genomes pathways enriched between high-risk group and low-risk group in the TCGA-COAD dataset.

The ssGSEA results suggested that the ssGSEA scores of iDCs, Inflammation-promoting, MHC_class_I, and Th2_cells were higher in the low-risk group, and the survival was better in the high-scoring group, which might be the reason why patients in the low-risk group had better survival (Figures 9A–G). In addition, risk scores were higher in the high microsatellite instability group than in the stable group, suggesting that defects in mismatch repair may be closely associated with the risk of colon cancer patients (Figure 9H). Notably, GO (Gene ontology) and KEGG (Kyoto Encyclopedia of Genes and Genomes) enrichment analysis based on the GSEA method suggested that many pathways and biological processes were different in the high- and low-risk groups, which may be the potential mechanism behind our constructed signatures (Figure 10).

## Discussion

Colon cancer is characterized by high relapse rate and poor prognosis, and accurate prediction of the prognosis of colon cancer patients is critical to guide their treatment. The construction of prognostic models with RNA processing factors may be an essential complement to predicting the prognosis of colon cancer. We analyzed the GSE39582 dataset in the GEO database and screened 80 prognosis-related RNA processing factors. These genes were highly exploratory and could separate colon cancer patients into two subtypes with significant differentiation, with lower survival in subtypes with a higher stromal score, immune score, and immune cell infiltration. Throughout tumor development and progression to metastasis, the immune system, stromal cells, and tumor cells have a close interaction. This complex interaction can both inhibit and promote tumor growth (Pancione et al., 2014; Vinay et al., 2015). In general, CD8^+^ cytotoxic T cells (CTL) and CD4^+^ helper T (Th)1 cells suppress cancer development through the production of interferons (IFN) and cytotoxins, but chronic inflammation that persists in colon cancer may override these effects and promote cancer development (Balkwill and Mantovani, 2001; Zamarron and Chen, 2011; Murata, 2018). There is also convincing evidence in animal tumor models and human cancers that the formation of an inflammatory microenvironment plays a vital role in the development and progression of CRC (Grivennikov et al., 2010). These results align with the previously mentioned subtype with a poorer prognosis in terms of a higher stromal score, immune score, and immune cell infiltration.

In further investigation, we finally identified 13 prognosis-related RNA processing factors to construct the signature. Our findings indicated that DEDD2, GTF2H5, SNRPA1, BICD1, CELF1, and CLK3 were prognostic risk factors for colon cancer, while ADAD1, CMTR1, HNRNPUL1, FTSJ3, WDR43, THUMPD3, SNRPF were prognostic protective factors. Among the prognostic risk factors, SNRPA1 was reported to be highly expressed in colorectal cancer tissues and promoted cancer progression through the regulation of PIK3R1, VEGFC, MKI67, and CDK1 (Zeng et al., 2019). V-ets avian erythroblastosis virus E26 oncogene homolog 2 (ETS2) is a protooncogene that regulates numerous cellular functions, including proliferation, apoptosis, differentiation, transformation, and migration, and is overexpressed in various human cancers, including CRC (Hsu et al., 2004). CELF1 was reported to promote proliferation, migration, and invasion of CRC cells *in vitro* and *in vivo* through upregulating ETS2 and induced resistance to oxaliplatin (Wang et al., 2020). GTF2H5, BICD1, and CLK3 have been reported to be oncogenes in ovarian cancer and hepatocellular carcinoma and are strongly associated with prognosis (Gayarre et al., 2016; Li et al., 2019; Jiang et al., 2020). We used random forest and found that BICD1 is the most critical gene for prognosis in signature, and there is high expression of BICD1 at bulk-RNA level based on TCGA dataset (Supplemental Figure S1). We then performed immunohistochemistry to compare the expression levels of BICD1 in normal and cancer tissues in colon cancer patients, but the results showed that BICD1 expressed negatively in both tissues (Supplemental Figure S2). As alterations in mRNA stability and/or translational efficiency are increasingly reported in colorectal cancer, we hypothesize that post-transcriptional alterations of BICD1 may be present in colon cancer tissues and are conducting related experiments. Among the protective prognostic factors, high levels of ADAD1 have been reported to be strongly associated with the prognosis of colon cancer patients, and its improvement of patient prognosis may be associated with CD4^+^ T cells (Yang et al., 2020). Arginine methylation is a post-translational modification required to maintain genomic integrity. Arginine methylation of HNRNPUL1 regulates the interaction with NBS1 and recruits it to sites of DNA damage; therefore, it is hypothesized that HNRNPUL1 plays an important role in the repair of DNA damage and suppresses tumorigenesis (Gurunathan et al., 2015). Other prognosis-related genes have been rarely reported in colon cancer and other cancers, so the mechanisms of these genes in colon carcinogenesis progression deserve further investigation as potential therapeutic targets.

Subsequently, we built a convincing column line graph including age, gender, TNM stage, and risk score. ROC curve analysis, C-index, and calibration curve validated the predictive ability of the model. It may enable clinicians to assess patient survival more accurately and effectively. Furthermore, we scored immune cells and immune function by ssGSEA. We found that the pro-inflammatory response was significantly upregulated in the high-risk group. Notably, in addition to tumor cell resistance to apoptosis and autophagy, necroptosis pro-inflammation is another mechanism of resistance to death (Niu et al., 2022). Unlike autophagy, tumor cells release cytokines into the tumor microenvironment after necrosis, and the cytokines recruit immune cells to clear the necrotic tissue, but immunosuppressive cells also promote the metastasis of tumor cells and enhance invasiveness. Immune evasion and inflammatory responses are hallmarks of tumors, and the interaction between immune responses and inflammatory responses also has a significant role in the progression of colon cancer (Vinay et al., 2015; Mortezaee, 2020). For example, TGF-β increases the risk of cancer associated with chronic inflammation in the intestine, while TGF-β also induces immune evasion through suppressing the function of many components of the immune system (Hong et al., 2010). This is consistent with our previous findings of poorer prognosis in the high immune score group and the immune cell infiltration group in disease subtypes. Thus, the interplay between chronic inflammation and immune evasion in colon cancer progression and the specific mechanisms should be taken into account.

We explored the potential mechanisms of 13-gene signature in colon cancer by GO enrichment analysis and KEGG pathway analysis and found that a large number of pathways differed in high- and low-risk groups. Among them, numerous pathways related to cell adhesion were enriched in the high-risk group, and cell adhesion plays an essential role in tumor cells leaving the primary site, surviving in the circulation, extravasating to distant organs for implantation and finally metastatic foci growth (Strilic and Offermanns, 2017; Laubli and Borsig, 2019). In addition, because cell adhesion receptors are linked to intracellular signaling pathways, interactions between tumor cells and other cells and the extracellular matrix regulate cell phenotype, proliferation, differentiation, and migration, ultimately leading to immune evasion and metastasis of tumor cells (Cooper and Giancotti, 2019). The poorer prognosis of patients in the high-risk group may be related to the enhanced adhesion of shed cancer cells which increases immune evasion and tumor metastasis, leading to poorer patient prognosis. Pathways associated with DNA replication and mismatch repair were more enriched in the low-risk group. Mismatch repair (MMR) excises base mismatches to ensure high genome fidelity and intact replication, and MMR defects and loss of MMR function play an essential role in the development and progression of colon cancer (Baretti and Le, 2018; Cortez, 2019). In contrast, patients in the low-risk group have a stronger DNA replication and mismatch repair capacity than those in the high-risk group, resulting in slower progression of colon cancer and a better prognosis. In addition, DNA mismatch repair (MMR) defects result in high mutational phenotypes due to frequent polymorphisms and single nucleotide substitutions in short repetitive DNA sequences, termed microsatellite instability (MSI) (Ozcan et al., 2018). This is consistent with the previous results of a higher risk score and worse prognosis in the high microsatellite instability group.

However, our study has some limitations. It was a retrospective study with data from the GEO and TCGA databases and lacked information such as treatment and relapse records. Our conclusions need to be validated by *in vivo* or *in vitro* experiments and prospective clinical studies.

## Conclusion

In summary, this study identified a 13-gene signature with prognostic value for colon cancer patients. The RNA processing factor may regulate the pathogenesis of colon cancer by mediating the immune evasion and mismatch repair pathway.

## Data Availability

The original contributions presented in the study are included in the article/Supplementary Material, further inquiries can be directed to the corresponding authors.
