# Supplementary material for: Application of RNA processing factors for predicting clinical outcomes in colon cancer
Source: Front Genet. 2022 Sep 23;13:979001. doi: 10.3389/fgene.2022.979001 (PMC9538339; doi:10.3389/fgene.2022.979001)
Supplement: Supplementary file 1 [file Presentation1.pdf]

Supplemental Material

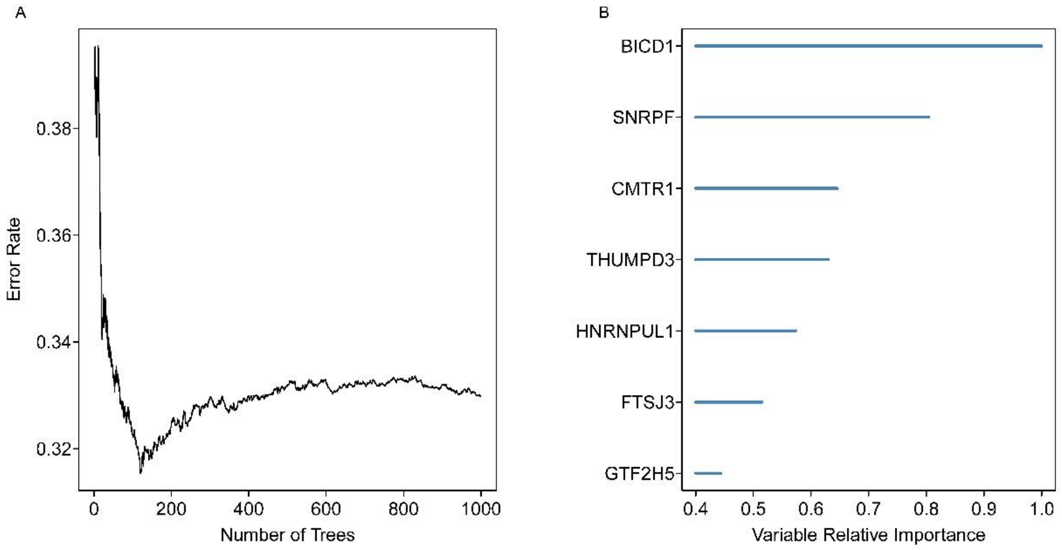

Supplemental figure 1. Kaplan–Meier overall survival analysis of IQGAPs mRNA expression in HCC patients from the TCGA datase. (1: IQGAP1, 2: IQGAP2, 3: IQGAP3, H: high expression, L: low expression, *p<0.05 for the comparison with All, #p<0.05 for the comparison with 1H 2L 3H).

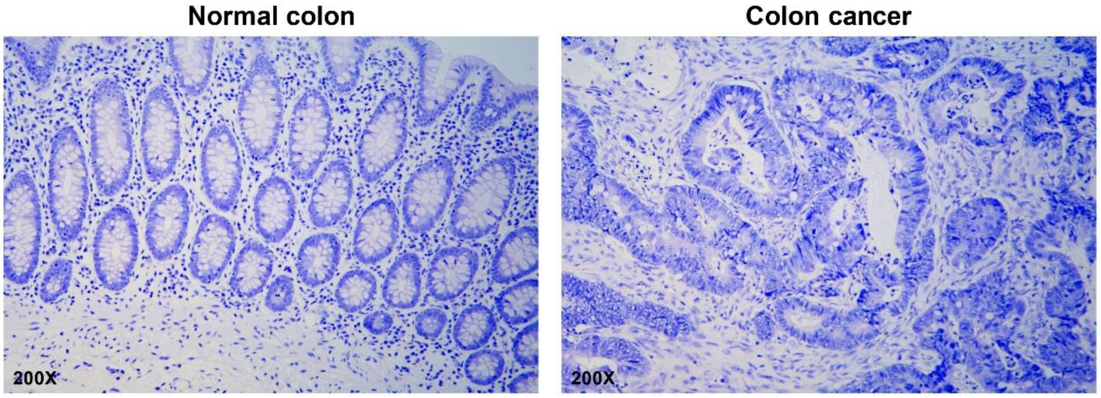

Supplemental figure 2. Representative images of BICD1 of normal and cancer tissues in colon cancer patients.
